# Supplementary material for: Empirical methods for controlling false positives and estimating confidence in ChIP-Seq peaks
Source: BMC Bioinformatics. 2008 Dec 5;9:523. doi: 10.1186/1471-2105-9-523 (PMC2628906; doi:10.1186/1471-2105-9-523)
Supplement: Additional File 1 — A variety of html documents from the USeq web site detailing the available applications, their best usage, output file type descriptions, command line menus, etc. [file 1471-2105-9-523-S1.zip › USeqUserGuides/JavaDoc.zip!/JavaDoc/edu/utah/seq/methylation454/class-use/Amplicon.html]

Uses of Class edu.utah.seq.methylation454.Amplicon


|  |  |  |
| --- | --- | --- |
| **Overview** \| **Package** \| **Class** \| **Use** \| **Tree** \| **Deprecated** \| **Index** \| **Help** | |  |
| PREV   NEXT | **FRAMES**    **NO FRAMES**     **All Classes** |  |


---


## **Uses of Class edu.utah.seq.methylation454.Amplicon**

| Packages that use Amplicon | |
| --- | --- |
| **edu.utah.seq.methylation454** | Applications for working with 454 data and identifying bisulfite converted bases for methylation studies. |

Uses of Amplicon in edu.utah.seq.methylation454

| Methods in edu.utah.seq.methylation454 that return Amplicon | |
| --- | --- |
| `Amplicon[]` | `Sample.getAmplicons()` |

| Methods in edu.utah.seq.methylation454 with parameters of type Amplicon | |
| --- | --- |
| `static java.util.ArrayList` | `StatMethylationData.findCommonCpGs(Amplicon one, Amplicon two)` |
| `void` | `Sample.setAmplicons(Amplicon[] amplicons)` |

---


|  |  |  |
| --- | --- | --- |
| **Overview** \| **Package** \| **Class** \| **Use** \| **Tree** \| **Deprecated** \| **Index** \| **Help** | |  |
| PREV   NEXT | **FRAMES**    **NO FRAMES**     **All Classes** |  |


---
